# Expression of *ADAMTS-8*, a secreted protease with antiangiogenic properties, is downregulated in brain tumours

**DOI:** 10.1038/sj.bjc.6603006

**Published:** 2006-03-28

**Authors:** J R Dunn, J E Reed, D G du Plessis, E J Shaw, P Reeves, A L Gee, P Warnke, C Walker

**Affiliations:** 1 JK Douglas Cancer Research Laboratories, Clatterbridge Hospital, Bebington, Wirral CH64 3JY, UK; 2 Department of Neurological Science, University of Liverpool, Lower Lane, Fazakerley, Liverpool, L9 7LJ, UK

**Keywords:** ADAMTS-8, glioma, brain tumours, angiogenesis, invasion

## Abstract

Angiogenesis and extracellular matrix degradation are key events in tumour progression, and factors regulating stromal–epithelial interactions and matrix composition are potential targets for the development of novel anti-invasive/antiangiogenic therapies. Here, we examine the expression of *ADAMTS-8*, a secreted protease with antiangiogenic properties, in brain tissues. Using quantitative RT–polymerase chain reaction (PCR), high, equivalent expression of *ADAMTS-8* was found in normal whole brain, cerebral cortex, frontal lobe, cerebellum and meninges. *ADAMTS-8* expression in 34 brain tumours (including 22 high-grade gliomas) and four glioma cell lines indicated at least two-fold reduction in mRNA compared to normal whole brain in all neoplastic tissues, and no detectable expression in 14 out of 34 (41%) tumours or four out of four (100%) cell lines. In contrast, differential expression of *TSP1* and *VEGF* was seen in nine out of 15 (60%) and seven out of 13 (54%) tumours, with no relationship in the expression of these genes. Immunohistochemistry and Western analysis indicated downregulation of ADAMTS-8 protein in >77% tumours. Methylation-specific PCR analysis of *ADAMTS-8* indicated promoter hypermethylation in one out of 24 brain tumours (a metastasis) and three out of four glioma cell lines suggesting an alternative mechanism of downregulation. These data suggest a role for *ADAMTS-8* in brain tumorigenesis, warranting further investigation into its role in regulation of tumour angiogenesis and local invasion.

The *ADAMs* and *ADAMTS* gene families encode related proteins characterised by an ADAM-like protease domain (*a d*isintegrin *a*nd *m*etalloprotease), and distinguished by one or more thrombospondin motifs (TS) in *ADAMTS* (review by Porter *et al*, 2005). The *ADAMTS* genes have varying functions, including inhibition of angiogenesis (*-TS-1*, *-TS-8*)(Vazquez *et al*, 1999), cleavage of the matrix proteoglycans aggrecan, versican and brevican *(-TS-1, -4, -5, -8* and *-15*)(AKA the ‘aggrecanases’)(Matthews *et al*, 2000; Collins-Racie *et al*, 2004; Porter *et al*, 2005), collagen processing (*TS-2, -3* and *-14*)(Colige *et al*, 1995; Colige *et al*, 1997; Fernandes *et al*, 2001; Wang *et al*, 2004) and blood coagulation homeostasis (*TS-13*)(Zheng *et al*, 2001). The ADAMTS proteins are secreted proteases, some of which bind to the extracellular matrix (ECM), unlike the ADAMs proteases that are mostly transmembrane proteins. ECM binding, substrate recognition and cleavage are mediated through the central and C-terminal TS repeats and the spacer region (Kuno and Matsushima, 1998; Tortorella *et al*, 2000).

*ADAMTS-8* and *TS-1* are the only two members of the *ADAMTS* family known to have antiangiogenic properties. They have been shown to specifically inhibit endothelial cell (EC) proliferation *in vitro*, suppress *FGF-2*-induced vascularisation in the cornea pocket assay, and inhibit *VEGF*-induced angiogenesis in the chorioallantoic membrane (CAM) assay (Vazquez *et al*, 1999). In addition, ADAMTS-1 can bind VEGF_165_, limiting its bioavailability; this binding is mediated by the C-terminal region of ADAMTS-1 (Luque *et al*, 2003). The function of VEGF on ECs is thus negatively modulated, and VEGF_165_, once bound, is unable to activate its receptor (VEGFR2).

In contrast to *ADAMTS-1*, *ADAMTS-8* has a narrow tissue distribution and human normal tissue showing moderate to high levels includes adult and foetal lung, aorta, brain, foetal heart, foetal kidney, appendix and bladder; both genes are expressed in adult human normal brain (Vazquez *et al*, 1999; Collins-Racie *et al*, 2004). There is evidence of modulation of expression of several of the *ADAMTS* genes in cancer, with significant downregulation (two-fold or lower) of *ADAMTS-8* in non-small-cell lung cancer (NSCLC) (Heighway *et al*, 2002; Dunn *et al*, 2004) and hypermethylation of the promoter region being a possible mechanism of gene silencing (Dunn *et al*, 2004). In comparison to non-neoplastic mammary tissue *ADAMTS-8* mRNA is also significantly downregulated in breast carcinomas (Porter *et al*, 2004). In contrast, *ADAMTS-4* (*aggrecanase-1*) and *TS-5* (*aggrecanase-2*) are upregulated in glioblastomas (GBMs), with a possible role in increased degradation of brevican thereby increasing invasive potential (Nakada *et al*, 2005; Held-Feindt *et al*, 2006).

Glioblastomas remain one of the most lethal neoplasms, due in part to their invasive nature and striking resistance to current therapies. There is currently a quest for a greater understanding of the biology of angiogenesis and invasion in gliomas with a view to the discovery of antiangiogenic/anti-invasive molecules that could be of therapeutic use in prolonging survival. ADAMTS-8 protein has a more powerful antiangiogenic effect than thrombospondin-1 (TSP1) or endostatin (Vazquez *et al*, 1999). The aim of this study was to investigate the expression of *ADAMTS-8* in high-grade gliomas and other brain tumours, and compare this with the expression of other well-characterised angiogenesis related genes *TSP1* and *VEGF*.

## MATERIALS AND METHODS

### Tissues and cell lines

Snap frozen tumour tissue for 41 resected brain tumours comprising 25 high-grade gliomas (20 glioblastoma WHO grade IV (GBM), two oligoastrocytoma WHO grade III (OAIII), one oligodendroglioma WHO grade III (OIII), one astrocytoma WHO grade III (AIII), one ependymoma WHO grade III (EIII)), five meningiomas WHO grade I, nine metastases, one haemangioblastoma, one medulloblastoma and one sample of non-neoplastic temporal lobe (from epilepsy surgery) were obtained for analysis from the Walton Centre for Neurology and Neurosurgery, Liverpool with full ethical approval. Intrinsic brain tumours were diagnosed according to the WHO histopathology classification (Kleihues and Sobin, 2000). For each case, one whole frozen tumour tissue fragment (∼0.5 cm^3^) was cut on a cryostat as follows: 10 × 30 *μ*M sections cut for extraction of RNA/DNA/protein, followed by a 5 *μ*M section for H&E staining and pathological examination. All tumours were confirmed to contain at least 70% neoplastic cells.

Normal brain total RNAs were purchased from BD Biosciences (whole brain), and AMS Biotechnology (cerebellum, cerebral cortex, cerebral meninges and frontal lobe). Normal brain DNAs from two separate donors were purchased from AMS Biotechnology. A non-neoplastic temporal lobe sample (from epilepsy surgery) was used in the Western and methylation analysis.

Four glioma cell lines U373, T98G, Hs683 and U87MG used in the RT–polymerase chain reaction (PCR) and methylation assays were obtained from ECACC and ATCC.

### Genomic DNA extraction

Genomic DNA was extracted from 10 × 30 *μ*M tissue sections using the Qiagen DNeasy™ Tissue kit according to the manufacturer's instructions.

Genomic DNA was extracted from cell lines using the Nucleon BACC1 kit according to the manufacturer's instructions.

### RNA extraction and cDNA synthesis

Total RNA was extracted from 10 × 30 *μ*m frozen tissue sections using the Qiagen® Rneasy Mini RNA extraction kit, following the manufacturer's protocol. RNA was aliquotted into 1–2 *μ*g amounts for subsequent Dnase treatment using Invitrogen Dnase I Amplification Grade. cDNA synthesis was performed in 20 *μ*l reactions using Invitrogen Superscript™ Reverse Transcriptase (RT), and oligo-dT primers (Invitrogen). For each sample, 500 ng of RNA was used for cDNA synthesis and the remaining 500 ng for a negative control (no RT). All new cDNA samples were diluted 20 × then 5 *μ*l of this dilution was amplified using cDNA specific PCR primers for the *β*-actin housekeeping gene to verify successful reverse transcription of sample cDNA with no genomic contamination (indicated by PCR product size).

### Quantitative (real-time) PCR

Quantitative PCR (qPCR) assay of *ADAMTS-8* expression in whole brain, cerebral cortex, frontal lobe, cerebellum, meninges and lung was undertaken to assess normal levels of the mRNA. Threshold cycles (*C*_t_s) were similar in all the brain regions tested therefore we used whole brain as the calibrator tissue in all subsequent qRT–PCR experiments. For comparison, the expression of *thrombospondin-1* (*TSP1)* and *vascular endothelial growth factor* (*VEGF)* was studied in a subset of cases (*n*=26, *n*=24, respectively).

To compare the relative expression levels of *ADAMTS-8*, *TSP1* and *VEGF* in normal whole brain to levels in multiple brain tumour tissues, we have used the comparative C_*t*_ method (Livak and Schmittgen, 2001) in which the expression of the test gene and a selected endogenous control gene (*Histone H3*) are measured in separate tubes. Normal whole brain cDNA was used as the calibrator tissue in each qPCR, which was carried out in an iCycler machine (BioRad). All PCR reactions were carried out in triplicate and each experiment was repeated at least once.

The threshold of detection for all primers was such that product could be detected from an initial RNA target of 50 ng in the RT reaction. Primer sequences, positions and efficiencies were as follows. *ADAMTS-8* primers spanned exons 3 and 4 (forward: 5′ AAC AAA AGC TGC TCC GTG AT-3′; reverse: 5′-TCT GGT TCA GGT GGA CGA AC-3′); *TSP1* primers spanned exons 22 and 23 (forward: 5′AGC AGG GTG CTA TTG TGA GG 3′; reverse: 5′CCT TAG TGC TTT GGC CTC TG-3′); *VEGF* primers spanned exons 3 and 4 (forward: 5′AGA AGG AGG AGG GCA GAA TC-3′; reverse: 5′ CAC ACA GGA TGG CTT GAA GA) to detect all *VEGF* isoforms; *Histone H3* primers spanned exons 3 and 4 (forward: 5′ CCACTGAACTTCTGATTCGC-3′; reverse: 5′ AAGACATCCAGCTAGCACGC-3′). Twenty microlitres of PCR reactions contained 5 *μ*l of 20 × diluted cDNA (equivalent to ∼6.25 ng of starting RNA), 10 *μ*l IQ™ SYBR® Green Supermix Buffer, 2 *μ*l forward and reverse primers (20 pmol/*μ*l) and 3 *μ*l ddH_2_O. cDNA was amplified under the following conditions: 95°C for 3 min, followed by 48 cycles of 94°C for 30 s, Tm (*ADAMTS-8*, *TSP1*=68°C, *VEGF*=64°C) for 30 s, 72°C for 30 s and a final step at 95°C for 30 s followed by collection of melt curve data under standard settings. Finally, 5 *μ*l of the resulting PCR product was visualised by electrophoresis through a 3% agarose gel containing ethidium bromide. Melt curves for the qPCR reactions and a single band of the correct size on the gels were used to verify the correct product in each PCR. In addition, the *ADAMTS-8* sequence in normal whole brain, lung and four brain tumours was verified by direct sequencing of the PCR product and BLAST analysis. Where mRNA expression data is described, downregulation has been designated 0.5 × or less, and upregulation as 2 × or more.

### Immunohistochemical analysis of ADAMTS-8

Immunohistochemistry (IHC) was performed as described previously (Dunn *et al*, 2004) using an ADAMTS-8 N-terminal antibody (1 : 500 dilution) (ADAMTS-8 AB-1, Oncogene Research Products; cat#PC508) and 5 *μ*m sections cut from formalin fixed, paraffin-embedded blocks from the same tumour resections. Each run included tissue sections from brain tumours, normal cerebellum and a positive control (normal human stomach) incubated with primary antibody, and a negative control with no primary antibody for each section. Protein expression was scored by a consultant neuropathologist (DDP) blinded to clinical details for each case; each section was assessed as to the type of cells staining, intracellular distribution and intensity of staining. Intensity was defined as negative (−), equivocal (+/−), or positive (with >10% neoplastic cells staining, +, ++ and +++). Downregulation of ADAMTS-8 compared to cerebellum is indicated by scores of −, +/− and +.

### Total protein extraction

A 4–5 mm^3^ piece of brain tissue was homogenised on ice in 100 *μ*l extraction buffer (1 M Tris-HCl pH 7.5, 1.5 M NaCl, 10% SDS, 5% NP40) containing one Protease Inhibitor tablet per ml buffer (Roche) using a micro-pestle. All homogenates were vigorously shaken at 3000 r.p.m. at room temperature for 30 min before centrifugation at 13 000 r.p.m. for 10 min. Protein supernatants were combined with laemmli sample buffer (Bio Rad) heated at 100°C for 10 min then plunged into ice immediately prior to gel electrophoresis.

### Western blot analysis of ADAMTS-8

Total protein extracts (SW480=6 *μ*g, non-neoplastic temporal lobe=10 *μ*g, tumours=30 *μ*g) were separated on 8% Tris-HEPES-SDS precast polyacrylamide gels (Pierce Precise™ Protein Gels, Perbio Science UK Ltd, Northumberland, UK). Each gel was divided into two halves (A and B), where both portions received an identical sample loading and where B subsequently served as a negative control blot, being incubated in blocking buffer without primary antibody. Gels were equilibrated in renaturation buffer (50 mM Tris-HCl pH 7.4, 20% glycerol) for 1 h prior to transfer. Proteins were then electrophoretically transferred to a nitrocellulose membrane, which was subsequently incubated overnight in blocker (PBS-T, 5% Marvel) at room temperature with shaking. Immunoreactive products were then detected by incubating for 1 h with ADAMTS-8 antibody (1 : 1000 dilution in blocker). Membranes were incubated for 1 h with a 1 : 3000 dilution (in blocker) of secondary antibody (HRP labelled anti-rabbit raised in donkey, Amersham NA 934VS), followed by chemiluminescent detection of specific proteins using Amersham ECL Advance kit and standard autoradiography procedures.

### Methylation analysis of the *ADAMTS-8* promoter region

One microgram of genomic DNA was chemically modified by treatment with sodium bisulphite using the CpGenome™ DNA modification kit from Intergen (Intergen Company, Oxford, OX4 4GA, UK, Catalog#S7820) according to the kit protocol. The modified, cleaned DNA was resuspended in 50 *μ*l TE buffer, aliquotted and stored at −80°C. A cMSP assay was used to assess the methylation status of the *ADAMTS-8* promoter region as described previously (Dunn *et al*, 2004). Polymerase chain reaction products (control: 299 bp, methylation specific: 169 bp) were analysed on 4% agarose gels containing ethidium bromide.

### Statistical analysis

Results are expressed as means. Statistical analysis was performed using SPSS (version 13.0). Differences between groups were evaluated by the Mann–Whitney test. mRNA and immunocytochemistry data were compared using Spearman's correlation test. A probability (*P*) value <0.05 was considered significant.

## RESULTS

### *ADAMTS-8* expression

#### mRNA

**Figure 1:**
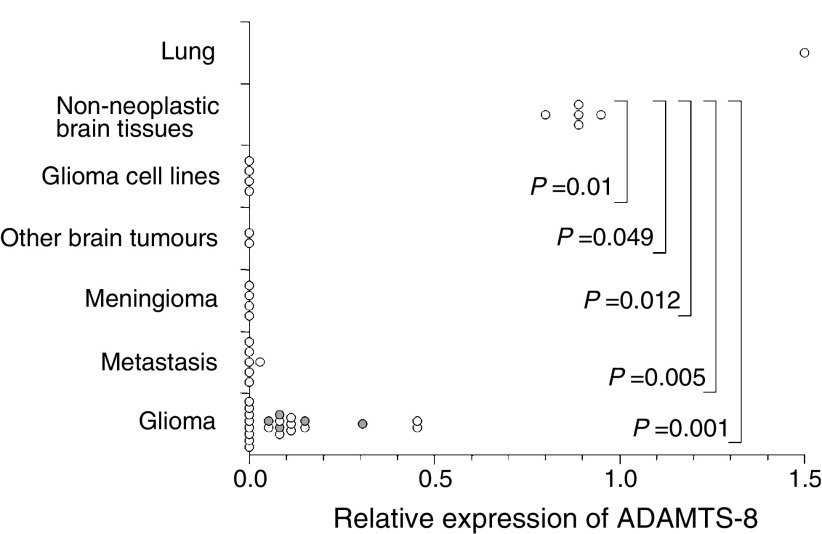
Expression of *ADAMTS-8* in brain tissues, glioma cell lines and lung relative to normal whole brain by quantitative RT–PCR. Grade III gliomas are indicated by shaded circles.

Non-neoplastic tissues: Gene expression analysis using RT–PCR showed equivalent levels of *ADAMTS-8* in normal whole brain, frontal lobe, cerebral cortex and meninges, with higher expression in normal lung (Figure 1).

Brain tumours: Hundred percent of cases showed lower expression of *ADAMTS-8* compared with normal brain. *ADAMTS-8* was not detectable in 14 out of 34 (41%) brain tumours, and downregulated between two- and a 1000-fold compared to normal brain in 21 out of 34 (62%) (Figure 1, Table 1). All gliomas showed at least two-fold downregulation of expression, with eight out of 22 (36%) tumours (all GBM) showing >40-fold downregulation (Figure 1). Only five grade III gliomas with varying histological subtypes were available for study, limiting comparison between different histopathological types of glioma. With the available series, GBM and grade III gliomas showed an overlapping range of relative expression of *ADAMTS-8* (GBM 0.0001–0.45; grade III gliomas 0.05–0.3)(Figure 1), and no significant difference between grades. When analysed separately, both GBMs and grade III gliomas showed a significant downregulation of ADAMTS-8 compared to the non-neoplastic brain tissues (Mann–Whitney test: GBM *P*=0.001, grade III *P*=0.009). *ADAMTS-8* was not detectable in three out of four meningiomas, four out of six metastases and four out of four glioma cell lines tested (Table 1). Despite the small number of cases, metastases and meningiomas were more likely to have lower *ADAMTS-8* expression than gliomas (Mann–Whitney test: metastases *P*=0.013, meningiomas *P*=0.031).

#### Protein

Immunohistochemistry: Immunohistochemistry analysis was carried out in formalin fixed, paraffin-embedded brain tissues comprising 35 brain tumours (25 gliomas (20 GBM, five grade III tumours), seven metastases and three meningiomas) and one normal cerebellum.

**Figure 2:**
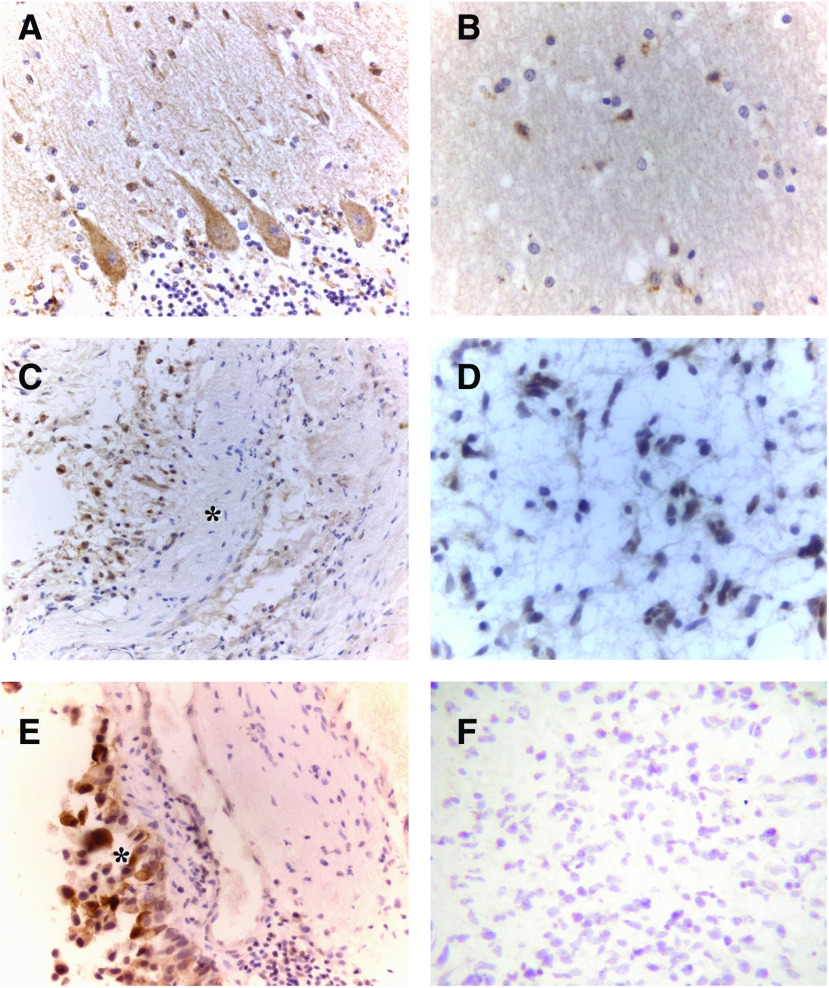
Expression of ADAMTS-8 protein by immunohistochemistry of formalin-fixed, paraffin-embedded brain tissues. (**A**) Normal cerebellar cortex (original magnification × 400). Positive cytoplasmic staining (++) of neurons in the molecular and Purkinje cell layers. (**B**) Normal cerebellar white matter (original magnification × 630). Positive cytoplasmic staining (++) of glial cells, most likely astrocytes. (**C**) and (**D**) GBM (case no.28). (**C**) (original magnification × 400) shows positive diffuse cytoplasmic staining (+/++) of tumour cells (left of image) adherent to a blood vessel (right side). The tumour cells show cytoplasmic expression and weaker, variable nuclear expression. The vascular endothelium shows weak expression and the vascular wall stroma (fibroblast and smooth muscle^*^) appears negative. (**D**) (original magnification × 630) shows malignant glial cells with cytoplasmic and some variable nuclear expression. (**E**) Metastatic carcinoma of gastrointestinal tract origin (case no. 25)(original magnification × 400). Strong focal cytoplasmic expression^*^ (+++) by malignant epithelial cells. Adjacent stroma contains fibroblasts (negative), lymphocytes (weak, variable staining) and vessels (with weak endothelial staining). (**F**) GBM case no. 6)(original magnification × 630). Negative staining of tumour cells.

Non-neoplastic brain tissues: Staining in normal stomach sections, which were used in this study as a positive control, was punctate and confined to the secretory type cells of the epithelial layer, as found in normal lung (Dunn *et al*, 2004). In normal cerebellum, the Purkinje and molecular cell layers showed focal cytoplasmic ADAMTS-8 staining of neurons (++) (Figure 2A, Table 1), and in the white matter, astrocytes were positive (++) for ADAMTS-8 protein (Figure 2B).

#### Brain tumours

In tumour sections, some staining was observed in reactive glia, vascular endothelium and some lymphocytes (Figure 2C and E). ADAMTS-8 protein in neoplastic cells was downregulated in the majority of tumours (27 out of 35, 77%) compared to cerebellum or uninvolved ‘normal’ non-neoplastic brain present within some tumour sections (Table 1). Most of the tumours (24 out of 35, 69%) showed some positive diffuse cytoplasmic staining for ADAMTS-8, with the exception of two mucinous-type metastases of gastrointestinal origin that showed focal punctate cytoplasmic staining similar to that seen in cerebellum, uninvolved brain, stomach and normal lung (Table 1)(Dunn *et al*, 2004). One tumour (GBM) was negative for ADAMTS-8 (Figure 2F, Table 1). Eleven gliomas (11 out of 24, 46%) showed variable nuclear staining (Table 1). Ten tumours scored +/−: three GBM, three grade III gliomas, three meningiomas and a non-mucinous type metastasis of breast origin (Table 1). Eleven GBM, two grade III gliomas and three metastases scored + (Table 1). Thus 70% GBM, 100% grade III gliomas, 100% meningiomas and 57% metastases showed decreased expression of the ADAMTS-8 protein compared to normal brain. There was no clear correlation between mRNA and immunopositivity (Spearman Correlation: all cases *P*=0.6, gliomas *P*=0.109). Eight tumours (eight out of 35, 23%), all with downregulated mRNA, showed upregulation or equivalent staining to that in uninvolved or non-neoplastic brain, including five GBM (with variable nuclear staining) and three mucinous type metastases of gastrointestinal origin (Figure 2C–E, Table 1). Of the tumours with no detectable mRNA, seven out of 11 were immunopositive. Overall, 27 out of 35 (77%) tumours including 20 out of 25 (80%) gliomas were considered to show downregulation of the protein.

#### Western blot

**Figure 3:**
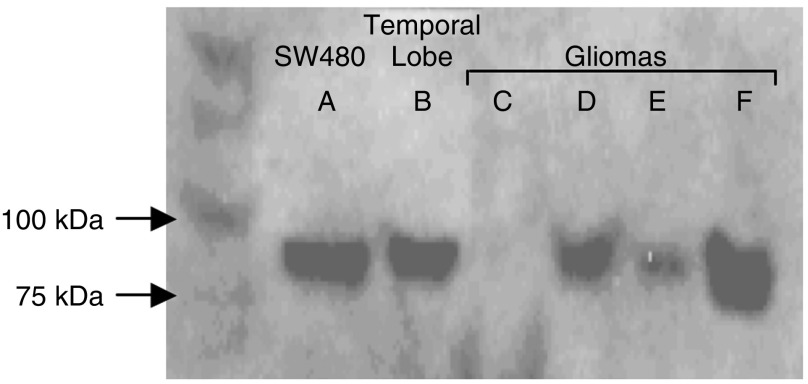
Chemiluminescent detection in Western analysis of full-length ADAMTS-8 protein (98 kDa) in total protein tissue extracts from normal temporal lobe and four GBMs, and commercial total protein extract from SW480 cell line (human colon carcinoma)(control). Protein loading amounts: SW480=15 *μ*g, normal brain=30 *μ*g, tumours=90 *μ*g.

Western blot analysis of the control SW480 cell line, non-neoplastic temporal lobe tissue and nine brain tumour total protein extracts (including five strong expressers of ADAMTS-8 by IHC analysis, see Table 1) using the same ADAMTS-8 primary antibody revealed one specific band at ∼98 kDa in the SW480 control, temporal lobe and three glioma extracts (all IHC positive) (Figure 3, Table 1). A protein loading of three times that of non-neoplastic brain was required to see any bands from the tumour extracts. The expected size for ADAMTS-8 using this antibody and the SW480 protein is 98 kDa. No specific bands were detected in the remaining tumour extracts (Table 1).

### Expression of *TSP1* and *VEGF* in brain tumours

#### mRNA

**Figure 4:**
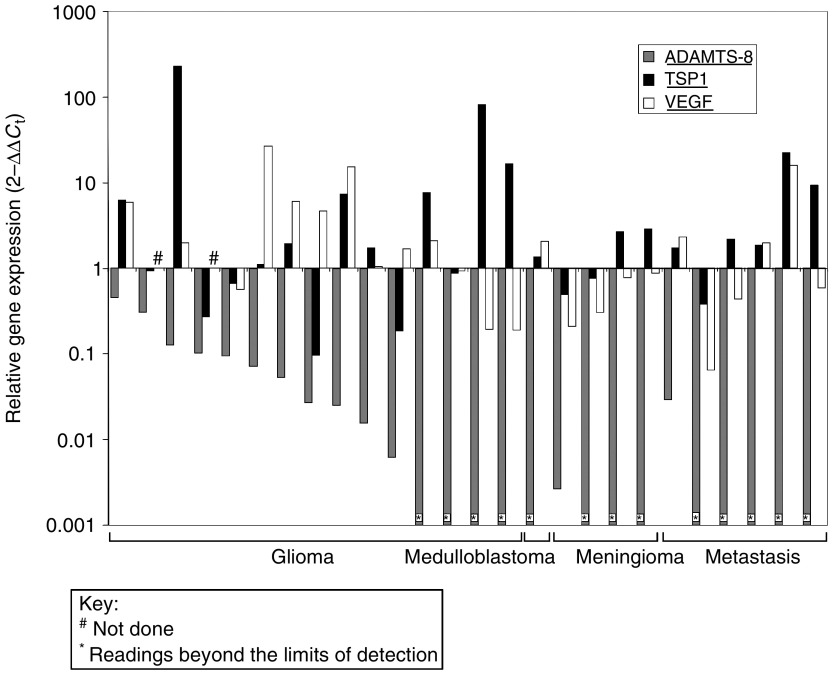
Expression of *ADAMTS-8*, *TSP1* and *VEGF* in brain tumours relative to normal whole brain by quantitative RT–PCR. *Y*-values are shown on a log scale where 1 signifies expression equivalent to that observed in normal whole brain.

Quantitative RT–PCR analysis showed that both genes were expressed in all cases, with both *TSP1* and *VEGF* being differentially expressed compared to normal brain in 62% (16 out of 26) and 63% (15 out of 24) of cases, respectively (Figure 4). There was no correlation between the expression patterns of *ADAMTS-8*, *TSP1* and *VEGF*. In summary, *TSP1* was upregulated in 42% (11 out of 26), downregulated in 19% (five out of 26) and unchanged in 38% (10 out of 26) of tumours tested. *VEGF* was upregulated in 38% (nine out of 24), downregulated in 25% (six out of 24) and unchanged in 38% (nine out of 24). In ten tumours that showed vascular proliferation typical of high-grade gliomas on examination of the H&E sections, nine also showed up-regulation of both *VEGF* and *TSP1*, accompanied by at least eight-fold downregulation of *ADAMTS-8*.

### *ADAMTS-8* promoter methylation status

**Figure 5:**
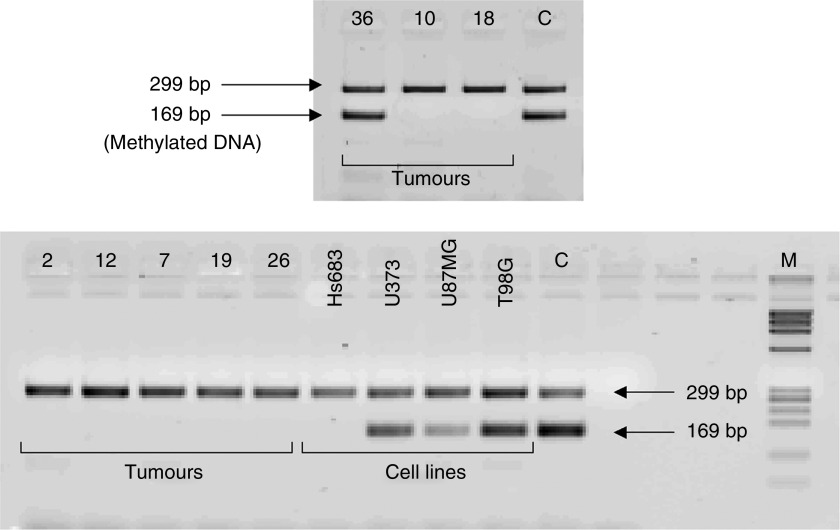
Competitive methylation-specific PCR (c-MSP) analysis of brain tumours and glioma cell lines. The presence of the methylation-independent control fragment (299 bp) confirms sufficient amounts of converted DNA in the reaction, while the presence of the methylation-specific product (169 bp) demonstrates the existence of methylated copies of DNA. DNAs from case 36 (metastatic carcinoma of GI origin) and glioma cell lines U373, U87MG and T98G showed methylation of *ADAMTS-8*, while cases 10 (OAIII), 2 (GBM), 12 (GBM), 7 (GBM), 18, 19 (meningiomas) and 26 (metastatic tumour of gastrointestinal/lung origin), and cell line HS683 showed no methylation. C, universally methylated DNA control; M, DNA molecular weight marker.

There was no evidence of methylation of the *ADAMTS-8* promoter in 23 out of 24 (96%) of the tumours, one out of four cell lines or in the three normal samples (Table 1). One tumour (a bowel metastasis) and three out of four glioma cell lines showed promoter methylation using this assay (Figure 5, Table 1).

## DISCUSSION

Brain tumours, especially GBM, have a propensity for diffuse infiltration of brain parenchyma that contributes to a high morbidity and mortality. The ability of tumour cells to infiltrate surrounding brain tissue is reliant on their passage through the ECM, facilitated in part by the actions of proteases. Similarly, angiogenesis (also essential to tumour progression) is reliant on the positive and negative regulation of proteases such as the *MMPs*, *ADAMs* and *ADAMTS* (review by Handsley and Edwards, 2005).

Until recently, the *ADAMs* and *ADAMTS* genes were relatively understudied in brain tumours (and other cancers), however, recent reports have suggested roles for *ADAMTS-4, TS-5* (Held-Feindt *et al*, 2006; Nakada *et al*, 2005) and *ADAM-12* (Kodama *et al*, 2004) in GBM, where these proteases are overexpressed. In contrast, we have shown that the expression of *ADAMTS-8* is downregulated in brain tumours relative to normal brain. Messenger RNA levels were reduced at least two-fold in 100% (34 out of 34) tumours and four out of four glioma cell lines tested, and protein data (IHC and Western) also suggested downregulation of ADAMTS-8. IHC analysis indicated decreased levels of protein in 77% of brain tumours tested compared to non-neoplastic tissue, and in Western blots, protein was undetectable in 67% of tumours and indicated a greater threshold of detection in 33%. Our data are consistent with other reports of *ADAMTS-8* downregulation in cancers (Heighway *et al*, 2002; Dunn *et al*, 2004; Porter *et al*, 2004), suggesting there may be opposing physiological roles for individual *ADAMTS* family members in carcinogenesis. For example the predominant role of *ADAMTS-8* may be antiangiogenesis, necessitating silencing in tumours, whereas the predominant role for *ADAMTS-4* and *TS-5* may be ECM degradation necessitating up-regulation. Studies of the *MMPs* have shown that some family members act as angiogenic molecules and some as antiangiogenic (review by Handsley and Edwards, 2005).

The antibody used in this study recognises a binding site within the prodomain and Western data revealed a 98 kDa ADAMTS-8 band common to both tumour and normal brain, consistent with the size of the full-length secreted protein after removal of the signal peptide. Using a cDNA probe containing a fragment of *ADAMTS-8* bearing a portion of the disintegrin domain and the first TS-type repeat, positive expression has been demonstrated in human normal whole brain, and various brain regions (Collins-Racie *et al*, 2004). However, other documented forms (79 and 64 kDa)(Vazquez *et al*, 1999) of ADAMTS-8, which retain antiangiogenic activity after proteolytic cleavage, may be present in normal and tumour brain tissues, would not be detected in the present study. ADAMTS-1 processing resulting in the release of the C-terminal end does not affect the catalytic function of the protease (Rodriguez-Manzaneque *et al*, 2000), with both processed forms having the ability to block EC proliferation in a dose dependent manner. Moreover, it is suggested that processing might release two fragments with distinct/independent functions in the ECM.

IHC data indicated the presence of ADAMTS-8 protein in the normal cerebellum and lower levels of protein in the majority (77%) of tumours tested (Table 1). Two mucinous type metastatic tumours of gastrointestinal origin showed strong staining (+++) for ADAMTS-8, although notably, neither of these cases gave a specific band in the Western analysis, which could be due to the lower proportion of ADAMTS-8 expressing cells within these samples. Most of the gliomas exhibited a diffuse cytoplasmic staining pattern and in addition 46% showed a variable nuclear stain. However, the Western data for two out of two samples with variable nuclear staining showed one band indicating specificity of the antibody at least under Western conditions. Overall the protein data for ADAMTS-8 suggest downregulation of the protein in the majority of brain tumours, although only further experimentation will determine the precise nature of this ADAMTS-8 protein.

Despite the downregulation of *ADAMTS-8* mRNA and protein seen in the majority of brain tumours studied, there was no clear correlation between protein and mRNA expression levels. All tumours had downregulation of mRNA, but this was associated with varying degrees of immunopositivity. These data may reflect the differing sensitivities of the RT–PCR and IHC assays, or suggest that *ADAMTS-8* expression may be regulated via transcription as well as factors that influence protein stability, with a longer protein half-life in some cases.

The mechanism of downregulation of *ADAMTS-8* in these brain tumours is unknown, but seems unlikely to be hypermethylation of the promoter region as observed in 58% (29 out of 50) of NSCLC (Dunn *et al*, 2004), since only one out of 24 tumours showed methylation using our assay. Notably, this tumour also showed hypermethylation in the promoter region of another gene situated within 20 kb on Chr11q25, and it is conceivable that this tumour contains an extensively methylated region of C11q25. C11q is not a region commonly showing deletion in LOH or CGH analysis in gliomas, however, there is evidence (from CGH analysis) of a 550 kb-deleted region at 11q13 in low-grade oligodendrogliomas (Rossi *et al*, 2005).

No correlation was found in the expression patterns of *ADAMTS-8, TSP1* and *VEGF*. It has been shown that *VEGF* is significantly overexpressed in GBMs (Plate *et al*, 1992; Shweiki *et al*, 1992), meningiomas, metastases and haemangioblastomas, however our data indicated up-regulation of *VEGF* in only 38% of tumours. Munaut *et al* (2003) found 2–15-fold upregulation of *VEGF* mRNA in most (17 out of 20) GBMs, downregulation in two and equivalent expression in one, although the relative abundance of isoforms 165, 121, 189 and 145 varied, with 165 being the most abundant. Since our *VEGF* PCR primers were designed to detect all isoforms, the relative abundance of *VEGF* isoforms in our samples was not assessed. However, if ADAMTS-8, like its counterpart TS-1, also binds VEGF_165_, ADAMTS-8 may sequester VEGF_165_, thus exerting an antiangiogenic effect. *VEGF* expression is upregulated in regions of hypoxia and localised to tumour vasculature, and in this study the expression of *VEGF* was seen in cases where a vascular proliferation was noted. *TSP1* was downregulated in the minority (19%) of the tumours tested and its expression did not correlate with that of *ADAMTS-8* (also antiangiogenic). It is known that although some genes involved in angiogenesis are segregated into proangiogenic or antiangiogenic, the real situation is far more complex. It has been shown that continuous expression of the antiangiogenic *TSP1* in brain tumours can induce a more aggressive angiogenic phenotype by inducing *VEGF* expression (Filleur *et al*, 2001; Filleur *et al*, 2003). In hepatocellular carcinoma, TSP-1 protein expression (*in vivo*) is significantly associated with tumour invasiveness and progression (Poon *et al*, 2004). Furthermore, in GBM, it has been demonstrated that tumour perfusion was the same in *TSP-1* transfected tumours and controls, and that although tumour growth *in vivo* is slowed by *TSP-1* overexpression, tumours still reach a large size (300 mm^3^)(Kragh *et al*, 2002). Moreover, like *VEGF*, the expression of *TSP1* was coincident with a vascular proliferation in nine out of 10 tumours. In hepatocellular carcinoma there is a significant relationship between tumour *TSP1* levels and venous invasion even in patients with high and low tumour *VEGF* levels (Poon *et al*, 2004). It is thought that *TSP1* expression in brain tumours is influenced by several factors including hypoxia, cell density (*in vitro*) and cell type (Tenan *et al*, 2000; Kawataki *et al*, 2000; Laderoute *et al*, 2000; Filleur *et al*, 2001; Naganuma *et al*, 2003).

The reduction in *ADAMTS-8* expression was not tumour type specific, since 22 gliomas, four meningiomas, six metastases, one haemangioblastoma and one medulloblastoma were analysed. However, as yet, the possible role of *ADAMTS-8* in brain tumour development remains unclear. There was no evidence to suggest that ADAMTS-8 expression is localised to the vasculature as for TSP1 and VEGF, or whether it is related to hypoxia. *ADAMTS-8* is downregulated in GBM, and further future investigation in low-grade and anaplastic gliomas that represent the various histological subtypes of glioma will reveal whether downregulation is an early event in gliomagenesis or, as for angiogenesis, may be associated with the transition from grade II to III.

In summary, the RT–PCR data suggest that in contrast to *TSP1* and *VEGF*, the downregulation of *ADAMTS-8* is a consistent event in the formation of brain tumours and is consistent with findings in other cancer types. Therefore, insight into its functional role and the biological events that regulate its expression is warranted. However, before we can ascertain whether *ADAMTS-8* and/or related molecules represent potential therapeutic targets in gliomas, further investigation of the *ADAMTS* gene family will be necessary to elucidate their potential roles relating to angiogenesis, ECM degradation and invasion.

## Figures and Tables

**Table 1:** Summary of ADAMTS-8 data in brain samples, showing case identifier, histology, expression of mRNA by qRT-PCR, expression of protein by immunohistochemistry and Western blotting, and the methylation status of the promoter region (methyl)

			**Protein expression**				
			**Immunohistochemistry**				
**Case**	**Histology**	**mRNA**	**C**	**NC**	**N**	**Western**	**Methyl**
w brain 1	Normal	=					
cerebel	Normal		++F				
temp lob	Normal	=				+	−
w brain 2	Normal						−
w brain 3	Normal						−
1	GBM	ND	+				
2	GBM	ND		++		+	−
3	GBM	Down			+/−		−
4	GBM	ND	+				
6	GBM	Down	−			−	−
7	GBM	Down	+				−
8	GBM	Down	+				
9	GBM	ND		++		+	
11	GBM	Down	+			+	
12	GBM	Down		+/−		−	−
13	GBM	Down	+				
15	GBM	ND		+			
28	GBM	Down		++			
29	GBM	Down	+				
30	GBM	Down	+				
31	GBM	Down		+			
33	GBM	Down	+/−				−
35	GBM			++		−	−
37	GBM			++			
39	GBM		+				
5	OAIII	Down	+				
14	OAIII	Down	+				
10	OIII	Down	+/−				−
27	AIII	Down		+/−			
32	EIII	Down			+/−	−	−
Hs683	Glioma CL	ND					−
T98G	Glioma CL	ND					+
U373	Glioma CL	ND					+
U87MG	Glioma CL	ND					+
34	haema	ND					
16	medul	ND					
17	mening	ND	+/−				−
18	mening	ND	+/−				−
19	mening	ND	+/−				−
20	mening	Down					
38	mening						−
21	met (lung)	Down	+				−
22	met (mel)	ND					−
23	met (br)	ND	+/−				−
24	met (k/pa)	ND	+				−
25	met (GI)	Down	+++F			−	−
26	met (GI)	ND	++				−
36	met (GI)		+++F			NS	+
40	met (lung)		+				−
41	met (lung)						−

Expression of mRNA data relative to normal whole brain and was equivalent (=), not detectable (ND) or downregulated (Down). Immunohistochemistry data show intensity of staining (− indicates negative; +/−, equivocal; +/++/+++, positive) the subcellular location of the stain (C indicates cytoplasmic; NC, nuclear and cytoplasmic; N, nuclear) and staining pattern, which was diffuse unless described as focal (F). Western data indicates the presence (+) or absence (−) of a 98 kDa band. Methylation data indicate promoter hypermethylation (+) or no methylation (−).

w brain=whole brain; cerebel=cerebellum; temp lob=temporal lobe; GBM=glioblastoma; OA=oligoastrocytoma; O=oligodendroglioma; A=astrocytoma; E=ependymoma; haema=haemangioblastoma; medul=medulloblastoma; mening=meningioma; met=metastatic tumour, followed by origin of primary tumour – br=breast; k=kidney; mel=melanoma; pa=pancreas; GI=gastrointestinal tract; NS=nonspecific bands.
